# A Survey on Wireless Body Area Networks for eHealthcare Systems in Residential Environments

**DOI:** 10.3390/s16060831

**Published:** 2016-06-07

**Authors:** Mohammad Ghamari, Balazs Janko, R. Simon Sherratt, William Harwin, Robert Piechockic, Cinna Soltanpur

**Affiliations:** 1 Department of Electrical and Computer Engineering, University of Texas at El Paso, El Paso, TX 79968, USA; 2 Department to Biomedical Engineering, University of Reading, Reading RG6 6AY, UK; b.janko@reading.ac.uk (B.J.); r.s.sherratt@reading.ac.uk (R.S.S.); w.s.harwin@reading.ac.uk (W.H.); 3 School of Electrical and Electronic Engineering, Bristol University, Bristol BS8 1UB, UK; r.j.piechocki@bristol.ac.uk; 4 Department of Electrical and Computer Engineering, University of Oklahoma, Norman, OK 73019, USA; cinna@ou.edu

**Keywords:** biomedical, eHealthcare, information and communications technology (ICT), telemonitoring, wireless body area network (WBAN), wireless technology

## Abstract

Current progress in wearable and implanted health monitoring technologies has strong potential to alter the future of healthcare services by enabling ubiquitous monitoring of patients. A typical health monitoring system consists of a network of wearable or implanted sensors that constantly monitor physiological parameters. Collected data are relayed using existing wireless communication protocols to a base station for additional processing. This article provides researchers with information to compare the existing low-power communication technologies that can potentially support the rapid development and deployment of WBAN systems, and mainly focuses on remote monitoring of elderly or chronically ill patients in residential environments.

## 1. Introduction

The ageing population around the world has been rapidly growing as a result of increased longevity, mainly attributable to the substantial improvement in nourishment, medicine and public health. In the United Kingdom alone, the population over the age of 85 is predicted to nearly triple by 2035 [1]; in the United States, the population over the age of 65 is estimated to double by 2040 [2]; in the People’s Republic of China, the population over the age of 60 is expected to double by 2040 [3]; and by the year 2050 Japan will have the eldest population in human history, with an average age of 52 years [4].

Simultaneously, public-funded healthcare systems in many developed countries are currently confronting an increase in the number of people diagnosed with chronic diseases such as obesity and diabetes. These chronic illnesses are not simply a result of ageing population but are due to inappropriate diet, sedentary lifestyle and insufficient physical activity [5,6]. As reported by the World Health Organization (WHO), diabetes is estimated to become the seventh leading cause of death by 2030 [7]. Due to its chronic nature, diabetes is an expensive illness not only for individual patients but also for healthcare systems as well.

These estimates and statistics indicate the fact that, continuously providing healthcare services to patients who are diagnosed with chronic conditions and increasing number of elderly people with various health difficulties is significantly increasing the cost of healthcare systems [8,9,10]. Therefore, healthcare systems are becoming unsustainable in their current form [9,11]. According to scientists [12,13,14,15], early disease detection and diagnosis is extremely important; on the one hand, it assists to effectively slow the progress of illness [13,14,15]; on the other hand, it helps to significantly reduce the cost of healthcare systems [12,14].

It is, however, possible to utilize the latest technological advances in Wireless Body Area Network (WBAN) systems along with Information and Communications Technologies (ICTs) for the early detection and prevention of potential diseases that may occur later in the people’s lives [16,17,18]. This can be done by integrating ultra-low-power none-invasive and/or invasive sensor nodes into WBAN systems for continuous monitoring of health conditions [19]. Each node within a WBAN system is capable of capturing physiological data such as electrocardiogram (ECG), electroencephalography (EEG), respiratory rate, body temperature and movement and transmits the collected data either as raw samples or low-level post-processed information to a base station wirelessly in order to be further analyzed and processed [20]. A WBAN system is able to provide long-term health monitoring of people without limiting their daily activities [21]. Such a system can be utilized to develop an intelligent and inexpensive healthcare monitoring solution which can be used as part of a diagnostic process [22]. The future system will be able to remotely monitor elderly people and chronically ill patients in their own residential environments where they are most relaxed and comfortable, and to minimize expensive hospitalization costs and reduce frequent hospital visits [22].

There are similar published studies in this area such as [23,24] that investigate some aspects of WBAN research such as physical and data link layer, and also compare a number of low-power radio technologies. The primary contribution of this paper is to not only investigate and compare the existing low-power on-body communication technologies, but also to consider the requirements and challenges of these low-power wearable technologies to communicate with the home infrastructure. Therefore, this paper considers the applicability and practical use of the existing low-power wearable technologies in a residential environment.

### 1.1. Residential Environment eHealthcare System Architecture

**Figure 1:**
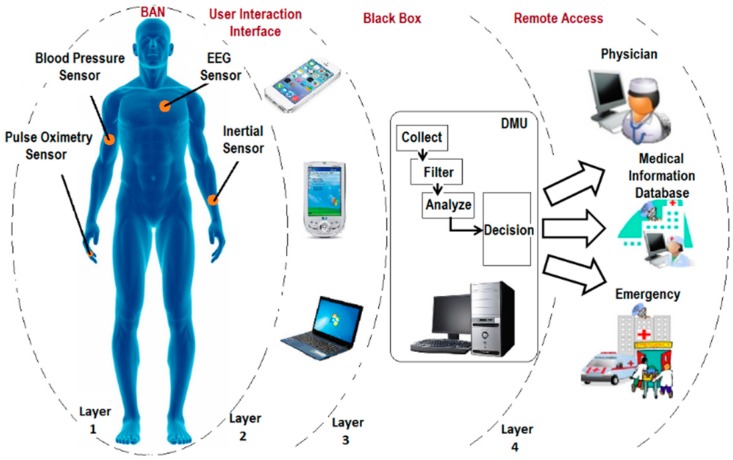
The four-layer typical architecture of an eHealthcare system.

A typical architecture of a residential environment eHealthcare system [25] consists of four layers as shown in Figure 1. Each layer of this architecture is further explained in more detail as follows. The BAN layer (layer 1) incorporates a number of sensor nodes operating within a wireless network. Sensor nodes in this layer are designed such that they can be placed on the human body as very small patches (on-body sensors), sewed into fabric (wearable sensors), or implanted under the skin (in-body sensors).

Such sensors continuously capture and relay vital parameters. However, depending on the functionalities and computation capabilities of nodes, data may require low-level on-tag processing prior to transmission. The collected data then may either initially be relayed to a central coordinator on the body or may be transmitted directly to the upper layers for further processing. The required transmission power by a sensor node in an off-body communication is mainly dependent on a number of factors such as Body Path Loss (BPL), Receive Noise Figure (RNF) and Signal to Noise Ratio (SNR) [26]. BPL greatly depends on the radiation patterns of the antennas used [27,28]. RNF is also a device-dependent factor. Each device has its own RNF and is indicated in its datasheet. SNR however is influenced by the quality of the overall communication link. The performance of SNR can be improved by a number of techniques such as Error Control Coding (ECC) techniques and Single-Input and Multiple-Output (SIMO) methods [29,30].

Layer 2 contains user interaction devices. Depending on the selected wireless communication protocol, different devices may be required to be used. For instance, Bluetooth-based sensor nodes require Bluetooth-based monitoring devices such as smartphones or PDAs. Layer 2 acts as an Access Point (AP). APs for residential monitoring are usually located within a room environment. Each room is equipped with an AP, where wireless devices are connected to a wired network, Wi-Fi or other relevant standards [31]. Collected data from this layer is required to be transferred to an upper layer (layer 3) in order to be prepared for the final destination. From room (layer 2) to black box (layer 3), there are a number of home networking possibilities that need to be considered [32].

There are three “room-to-box” scenarios which are explained in more detail as follows. First scenario provides an approach based on dedicated cabling. In this scenario, either both data and power are transferred over a cable (e.g., Power over Ethernet (POE)) [33] or data and power are transferred over separate cables (e.g., power over mains and data over Ethernet) [34]. The main disadvantage of this scenario is the requirement for cable installation which adds repetition complexity and cost to the system [35]. Table 1 lists some of the existing wired home networking technologies that can potentially be used to transfer the data over the cables [35,36].

The second scenario relies on Power Line Communication (PLC) technology, where data and power are transferred over the mains [37]. The main advantage of this scenario is the use of existing electrical wiring infrastructure and electrical outlets [38]. PLC is a reliable technology and in terms of cost, it is less expensive than a dedicated cabling scenario [38]. However, embedded based standards for PLC are limited in bandwidth. Another important disadvantage of PLC technology is that data may be lost due to an unexpected power outage [38].

The final scenario is based on existing wireless communication protocols such as Wi-Fi or ZigBee in order to transfer the collected data from rooms to black box [35]. However, this communication method is considered less reliable when it is compared to dedicated cabling and PLC technology [38].

The third layer of the proposed system architecture as depicted in Figure 1 consists of a Decision Measuring Unit (DMU). A DMU is an automatic computing system which performs all major computing operations and is connected to the Internet. It is the main core of the solution where all important decisions are made. The role of the DMU is to collect, filter and analyze the information. The aim of the DMU is to create a typical example of resident’s environment that includes a comprehensive database of resident’s medical profile. The DMU is able to recognize resident’s conditions based on the information obtained from a number of sensors which are transformed into knowledge and a list of user-defined policy rules. Subsequently, appropriate decisions are made automatically regarding the health status of inhabitant. The DMU is connected to a back-end medical institution such as a hospital in which physicians are able to consider people’s health status.

The last layer (layer 4) of this architecture as shown in Figure 1 provides healthcare services to patients. The analyzed data stored in the DMU is delivered to a remote server in a hospital, where medical professionals have access to it. In this layer, two different types of services may be provided by healthcare personnel: healthcare services and emergency services.

### 1.2. Taxonomy and Requirements

Let us summarize the primary requirements and design considerations of wireless communication technologies that can potentially be applied in WBAN systems before ending this section. These requirements can be categorized into four main subjects: low power consumption, transmission reliability and latency, data rates, security and privacy.

#### 1.2.1. Low-Power Consumption

Low-power consumption is considered to be one of the most important and challenging requirements in WBAN systems. Devices in WBAN systems mainly consume energy during sensing vital information, wireless communication and data processing. However, compared to sensing information and data computation, wireless communication consumes a significant amount of energy. Thus, reducing the energy consumption of data transmission during communication can conserve considerable amounts of the energy reserves. In almost all WBAN devices, batteries are the main source of power supply, but they are also the largest component in terms of weight and volume compared to other electronic components. This is important because in many WBAN applications such as pacemakers, wearable devices must be able to operate for very long duration of time without being recharged or replaced. Many techniques have been proposed in the past to lower the power consumption of such devices. As an example, an energy-efficient hybrid system has recently been proposed by Ghamari *et al.* [39] to minimize the required transmission energy consumption of such systems by utilizing energy harvesting techniques and low-power MAC protocols. In order to minimize power consumption, it is also important that the upper layer, the application layer, uses a better strategy of sampling and transmitting data that is more convenient for its application. As an example, the system can reduce the sampling rate of pulse when the user is at rest according to the motion sensor. Dieter *et al.* [40] and Krause *et al.* [41] showed how selective sampling strategies can decrease the power consumption of such systems which results in an increase in the deployment lifetime of wearable technologies. Furthermore, authors in [42] believe that, in order to lower the power consumption, it is also possible to reduce the sampling rate below the Nyquist rate while still achieving an acceptable quality reconstruction.

In the bigger picture, the topology of network and placement of sensors also play a role in power consumption. The network topology consists of multiple healthcare sensors and relay nodes. Location of these network elements are mostly fixed and are in close proximity of each other. In this setup, the messages can be relayed through the network to communicate various physiological parameters. The optimization of network mesh and positioning is explained in [43]. Authors in [44,45] show that cooperative transmission with the use of relay nodes can boost the efficiency. In [46], Ahmed *et al.*, refined the network/MAC layer towards an energy efficient routing protocol. A joint optimization of positioning and routing is developed in [47]. Finally, in [48] Liu *et al.* considered the quality of service (QoS) requirements.

#### 1.2.2. Transmission Reliability and Latency

Data transmission reliability and latency are two extremely important factors in patient monitoring applications. High reliability and low latency of data transfer ensures that real time data is successfully transmitted and is immediately accessible to healthcare providers. Reliability directly influences the quality of patient monitoring. It can be life-saving in many situations and in a worst-case event; it can be disastrous when a life threatening incident has not been observed or detected.

On-body channel modeling is another key consideration that has significant impact on the robustness of the communication link. On-body radio propagation channels are mainly influenced by the frequent body movements and dynamic characteristic of the communication channel. Although complicated analysis techniques such as Finite-Difference Time-Domain (FDTD) is able to provide an accurate representation of static on-body radio propagation as shown in [49], extending such analysis into dynamic on-body channel modeling cases is typically too costly. As a result of that many studies focus on statistical techniques or uncomplicated analytical approaches [50,51].

In addition, data transmission reliability and latency are mostly relied on the design of Physical (PHY) and Medium Access Control (MAC) layers. In order to achieve optimal reliability and network efficiency, appropriate MAC layer protocols are required to be designed to fulfill the particular needs of specific applications [37,38]. Reliability of WBAN systems can also be determined in terms of their major Quality of Service (QoS) parameters such as transmission loss rate, delay profile and delay jitter.

#### 1.2.3. Data Rates

Due to the great diversity of the applications in WBAN systems, data rates differ greatly, ranging from low data rate sensors focused mainly on on-body monitoring at a few kbps to high data rate systems designed for multimedia data streams of several Mbps [52]. Information may also be transmitted in bursts, though this way of transmitting information is not considered energy efficient due to the fact that burst transmission sends out very high data transmission rate with very short transmission durations. In medical applications, the reliability of the WBAN systems also depends on the employed data rates as low data rate devices are able to manage high BER environments, whereas, devices with higher data rates are most suitable to be used in lower BER conditions [53].

#### 1.2.4. Security and Privacy

The transmission of health-related information between on-body sensors and monitoring devices in WBAN systems and subsequently over the internet to central controllers in hospitals is strictly private and confidential. Health-related information must be encrypted so that the patient’s privacy is protected. Healthcare professionals who have access to information must be confident that the patient’s vital information is not tampered with or altered and did truly originate from the monitored individual. Furthermore, an overly secure system might disallow healthcare professionals from accessing vital health-related information in certain emergency events and thus jeopardize patient’s life. Moreover, enriching the current systems with security and privacy mechanisms significantly increases the cost of energy for communication which results in more power drain from small batteries [52].

The rest of this paper is organized into six sections. Section 2 reviews a number of existing low-power communication technologies that are appropriate candidates for remote health monitoring applications. Section 3 compares and discusses the advantage and disadvantage of using the existing low-power technologies. Section 4 discusses the future prospects of remote health monitoring systems. Section 5 provides a brief overview of some of the most recent research articles published in the area of telemonitoring systems and finally Section 6 provides a conclusion to this article.

## 2. Candidate Wireless Technologies

This section reviews the latest wireless communication technologies that are able to support the rapid development and deployment of BAN systems.

### 2.1. Popular Low-Power Wireless Technologies

#### 2.1.1. Bluetooth Low Energy (BLE)

As part of the Bluetooth 4.0 standard, an alternative to classic Bluetooth known as Bluetooth Low Energy (BLE) was introduced [54]. BLE was initially developed by Nokia in 2006. It was designed to provide an extremely low power idle mode, uncomplicated device discovery and highly reliable transfer of data. BLE is able to wirelessly connect miniature, low-power devices to mobile terminals which make it an appropriate candidate for the health-monitoring (BAN) applications. BLE is hardware-optimized version of Bluetooth because of its main differences such as data packet format, radio transceiver and baseband digital signal processing compared to classic Bluetooth. BLE is able to provide up to 1 Mbps data rate. Since BLE utilizes fewer numbers of channels for pairing BLE devices, it consumes considerably less time (few milliseconds) for device discovery and synchronization compared to seconds for Bluetooth. This is significantly valuable for resource-limited and latency-critical devices such as those used in health-monitoring applications. BLE employs a simplified protocol stack and is mainly concerned on short-range, star-topology network with uncomplicated routing algorithms.

#### 2.1.2. IEEE 802.15.4 and ZigBee

IEEE 802.15.4 [55] and ZigBee [56] are two widely used radio standards in BAN applications. IEEE 802.15.4 technology includes physical (PHY) and Medium Access Control (MAC) layer protocols focusing on low data rate and medium-range wireless communications which makes it an appropriate solution for health-monitoring applications.

Similar to IEEE 802.15.4, ZigBee is an enhanced version which provides additional layer protocols such as network, security and application layers that reside on top of physical and MAC layers defined by IEEE 802.15.4. The main purpose of both standards is to provide low power solution for battery-powered devices. The physical layer utilizes Direct Sequence Spread Spectrum (DSSS) modulation technique for interference mitigation and the MAC layer also utilizes Carrier Sense Multiple Access with Collision Avoidance (CSMA/CA) for channel access. The ZigBee standard provides support for flexible network topology. Devices in a ZigBee network are distinct between Reduced Function Device (RFD) and Full Function Device (FFD). FFDs are able to set up a mesh network where low duty cycle reduced function devices join the network as leaf nodes. In addition, ZigBee fully supports the low duty cycle operation of nodes (sensor nodes turn off their radios most of the time to reduce energy expenditure).

Contrary to ZigBee, classic Bluetooth does not support low duty cycling operation. In Bluetooth devices, a slave node must be kept synchronized to the master node for data transmission. As a result of that, there is an increase in ‘radio on’ period which in turn leads to increased energy consumption. The ZigBee Alliance incorporates several public profiles which simplifies distribution of systems with interoperable multi-supplier ZigBee-based devices. For instance, ZigBee has recently developed a profile termed Personal Health and Hospital Care (PHHC) [57]. The aim of this profile is to provide reliable and secure monitoring of non-invasive, non-critical healthcare applications mainly focused on physical fitness, chronic disease and aging. The PHHC profile also fully supports ISO/IEEE 11073 standard [58] and utilizes the ISO/IEEE 11073 protocol for exchange of medical information.

Moreover, the ZigBee Alliance has recently introduced an optional feature in the ZigBee 2012 specification. This feature is termed ZigBee PRO Green Power [59]. Green Power provides adequate power for battery-less devices with harvested energy and allows them to actively join ZigBee PRO 2012 networks. Furthermore, IEEE 802 has recently introduced the first international WBAN standard called IEEE 802.15.6 [60]. The main purpose of this standard is to provide a short-range, low-power and extremely reliable communications in the vicinity of or inside the human body. IEEE 802.15.6 is targeted to serve a range of medical and non-medical applications.

### 2.2. Alternative Low-Power Wireless Technologies

#### 2.2.1. Classic Bluetooth

Classic Bluetooth is a Wireless Personal Area Network (WPAN) technology [61] where a number of Bluetooth devices (up to eight) form a short-range personal network known as a piconet. In Bluetooth, slave devices must be paired and synchronized with a master device before data communication starts. This is usually achieved via the use of common clock between the communicating devices. Bluetooth operates in the 2.4 GHz ISM frequency band; it utilizes frequency hopping mechanism among 79 channels with an average rate of 1600 hops per second to minimize interference. The Bluetooth standard classifies devices into three groups based on transmission power and corresponding coverage area. The wireless communication range supported by the standard provides adequate coverage for WBAN communications. The Bluetooth SIG has developed the Health Device Profile (HDP) [61,62] capable of providing usage models for fitness and healthcare devices. HDP is also able to wirelessly connect devices such as glucose meters, pulse oximeters, weight scales, thermometers and blood pressure monitors to sink devices such as cell phones, PDAs, laptops and personal computers.

#### 2.2.2. ANT

ANT [63] is a low-power proprietary wireless technology designed and developed for a broad range of wireless sensor network (WSN) applications. ANT is specifically appropriate for low data rate battery powered sensor nodes and covers a range of network topologies from simple peer-to-peer to complex mesh networks. ANT is a candidate for wireless connectivity in battery powered applications such as health monitoring where ultra-low power consumption is required. ANT operates in the 2.4 GHz frequency band, supports a data rate of 1Mb/s and employs TDMA scheme to address interference issues. ANT+ facilitates wireless communication of devices from different companies by providing predefined network parameters and data payload structures including device profiles. Existing ANT+ device profiles consist of heart rate monitors, stride-based speed and distance monitors, bike speed and power. Several upcoming device profiles include weight scales, multi-sport speed and distance, and environment sensors.

#### 2.2.3. RuBee

RuBee [64] is considered an alternative to Radio-Frequency Identification (RFID). It is a bidirectional active wireless protocol that employs long wave magnetic signals (not RF signals) to transmit and receive 128 byte packets of data within a local network. RuBee is based on the IEEE 1902.1 [65] standard and is specifically designed to provide high security in harsh environments. Similar to the IEEE 802 standards, RuBee enables the networking of devices by employing point-to-point active radiating transceivers. This protocol operates at the low frequency end, 131 kHz. Similar to WiFi, Bluetooth and ZigBee, RuBee is an on-demand packet based protocol but with lower data rate. In addition, RuBee’s low operating frequency provides a significant benefit in terms of power consumption. It can provide a battery life of up to fifteen years using a single lithium button cell battery and it is also able to provide a coverage distance of up to 50 feet according to [58]. However, RuBee’s low operating frequency requires a bigger antenna size which makes this technology a likely inappropriate candidate for BAN applications where the size of antenna plays an important role. In contrast to RFID, RuBee does not have signal reflections and cannot be blocked by materials such as steel and liquid. Therefore, it is a robust technology especially in harsh environment visibility and security applications [66,67].

#### 2.2.4. Sensium

Sensium [68] is an ultra-low power wireless platform mainly designed to provide customized health services for chronic disease management applications [69]. Sensium is capable of providing an ultra-low power monitoring of vital health signs such as PH levels, blood glucose and ECG signals. The main aim of Sensium is to be embedded in a digital plaster to be prescribed by physicians. Sensium operates in the 900 MHz frequency band and supports a data rate of 160 kb/s. Sensium is considered as one of the leading ultra-low power wireless technologies for low data-rate on-body applications. Sensium utilizes a master/slave communication structure, in which an on-body slave node transmits multiple vital signs to a personal server such as smart phone, PDA or a personal computer from time to time. Since sensium utilizes a star topology, joining a network is managed centrally. Energy consumption of nodes is also managed centrally; nodes are programmed to keep their radios in sleep mode until they are given time slots by central server.

#### 2.2.5. Zarlink

Zarlink [70,71] is a proprietary ultra-low power RF transceiver specifically designed for medical implantable applications. Zarlink utilizes Cyclic Redundancy Check (CRC) error detection along with Reed-Solomon error correction scheme to provide a highly reliable communication link. It operates in the MICS (402–405 MHz) and ISM (433–434 MHz) bands. Zarlink supports data rates of up to 800 kb/s. Zarlink is able to operate in both an implant and a base station. Depending on the selected system type, different requirement is needed especially in terms of power consumption. Therefore, Zarlink has specified two important operation modes: Implantable Medical Device (IMD) mode and base mode. When Zarlink is configured as an IMD mode, the radio is asleep most of the time which consumes only µW of power compared to mW of power in other modes. However, communication between two nodes cannot occur if either node’s radio is in sleep mode. Therefore, a mechanism is required to wake up the receiver’s radio to ensure the sender’s transmit and receiver’s listen operations coincide. This can be done by either utilizing an ultra-low power 2.4 GHz radio or directly by using the IMD processor. Zarlink is considered as one of the leading ultra-low power wireless technology for low-data rate medical implantable applications (TRX = 5 mA, low-power mode = 1 mA and ultra-low power wakeup circuit = 250 nA).

#### 2.2.6. Z-Wave

Z-Wave [72] is a proprietary wireless communication protocol mainly designed for automation in home and light commercial environments. Z-Wave was initially developed by ZenSys (now a division of Sigma Designs) [73] and is currently managed by the Z-Wave Alliance [74]. One of the main advantages of Z-Wave compared to some other technologies is that it operates in the sub-1 GHz band (around 900 MHz) which avoids interference with other wireless technologies operating in the crowded 2.4 GHz band such as WiFi, Bluetooth and ZigBee. Z-Wave technology utilizes a number of low-cost low-power RF transceiver chips which are embedded into home electronic devices such as lighting, intercom and entertainment systems. Z-Wave uses a low-power wireless technology to communicate with Z-Wave-based devices. This technology is optimized to provide reliable transmission of small data packets from a control unit to Z-Wave devices in a network. Z-Wave protocol utilizes frame check sequence, frame acknowledgement, retransmission, CSMA/CA and complex routing algorithms to ensure reliable communication in multipath environment of a residential house. Z-Wave supports mesh networking, provides 9.6 kb/s and 40 kb/s data rates, and uses Gaussian Frequency-Shift Keying (GFSK) modulation scheme. Z-Wave recently introduced the Z-Wave 500 series, a next generation upgrade to the Z-Wave chip and module which supports a higher data rate of up to 100 kb/s. Z-Wave is able to include up to 232 nodes in a Z-Wave network. This technology defines two different types of devices: controllers and slaves. Controllers have unidirectional control over slave devices. They are responsible for sending commands to slave devices, which receive the task and send back the corresponding answer [75].

#### 2.2.7. Insteon

Insteon [76] is a proprietary mesh networking technology specifically designed for home and personal electronics applications. Insteon makes use of both Radio Frequency (RF) signals and home’s existing electrical wiring infrastructure (PLC) to transmit data from one device to another. Insteon is able to utilize RF-only devices, power-line-only devices or can simultaneously support both types of communication systems. Therefore, it is considered as one of the most reliable home automation technology. Insteon devices are called peers because all Insteon devices are able to transmit, receive and relay other messages completely independent of a controller. Insteon communication range can be extended by means of a multi-hop approach. In this method, an Insteon network uses two or more hops to deliver information from a source to a destination. Similar to Z-Wave, Insteon also limited the maximum number of hops allowed for each message to four. In addition, in PLC applications, Insteon operates at 131.65 kHz and uses Binary Phase-Shift Keying (BPSK) modulation technique; in RF applications it operates in the ISM (902–924 MHz) band and uses Frequency-Shift Keying (FSK) modulation scheme. Insteon utilizes Automatic Repeat request (ARQ) scheme to achieve reliable data transmission over unreliable or noisy communication channels. Insteon supports instantaneous data rates of 13.165 kb/s. It also supports a number of encryption methods such as rolling-code, managed-key and public-key [77].

#### 2.2.8. Wavenis

Wavenis is a wireless protocol architecture created as a proprietary technology by Coronis systems and promoted by the Wavenis Open Standard Alliance [78]. Wavenis is specifically designed to provide an ultra-low power and long-range wireless solution for a vast range of Machine to Machine (M2M) applications such as industrial process control, environmental monitoring and healthcare monitoring. In the majority of M2M applications, devices are expected to have low data rates and to operate on battery. However, recharging or replacing batteries not an easy task in many situations, saving battery power without compromising reliability is an important challenge. Moreover, a high link budget is needed to achieve adequately long-range communication in a number of M2M applications. Wavenis is an appropriate candidate to provide solution for these challenges. The main features of Wavenis technology include power conservation, reliability, network coexistence and resistance against interference. Wavenis operates worldwide in the 433 MHz, 868 MHz and 915 MHz ISM bands. It supports different data rates of 4.8 kb/s, 19.2 kb/s and 100 kb/s, uses GFSK modulation scheme and employs fast Frequency-Hopping Spread Spectrum (FHSS) technology. The MAC layer of the Wavenis protocol consists of two transmission techniques: synchronous and asynchronous. In the synchronous communication networking mode nodes are equipped with a combination of CSMA and TDMA channel access schemes. In this case, a randomly computed time slot is allocated to a node willing to acquire the channel. Prior to transmission in the allocated time slot, the node listen to the shared medium to check for any on-going transmission. If the shared medium is occupied by other nodes, the node calculates a new time slot for its next transmission. However, asynchronous communication networking applies in applications where reliability plays an important role such as security systems and in such applications CSMA/CA mechanism is used [75].

#### 2.2.9. BodyLAN

BodyLAN is an ultra-low power, low-cost and reliable BAN platform created as a proprietary technology by FitLinxx [79]. BodyLAN is designed to be used in a vast variety of applications such as consumer electronics, activity and wellness devices, medical devices and fitness equipment. In terms of power usage, BodyLAN provides much lower power consumption rate compared to Bluetooth devices. This wireless technology uses a single radio channel, short burst duration and extremely low duty cycle. BodyLAN utilizes GFSK modulation technique which prevents BodyLAN packets from colliding with 802.11 g/Orthogonal Frequency-Division Multiplexing (OFDM)/ DSSS packets. BodyLAN operates in the 2.4 GHz ISM band and supports data rates of 250 kb/s and 1 Mb/s. It also utilizes a peer-to-peer network topology without centralized timing. Devices in a BodyLAN network are categorized into two groups of transmit-only and transmit/receive devices. In terms of security, BodyLAN encrypts frame payloads and dynamically changes algorithms based on device addresses and timing plans. In addition, following the collection of data, the ActiHealth network utilizes a secure VPN connection between the ActiHealth data server and application servers in order to guarantee the security of the collected information.

#### 2.2.10. Dash7

Dash7 [80,81] is a proprietary open source, ultra-low power and long-range wireless communication protocol which was initially designed for military usage and has been adapted for use in commercial applications. Dash7 technology is based on the ISO/IEC 18000-7 open standard using an active RFID. This technology is currently managed by the Dash7 Alliance which offers interoperability among Dash7-based devices. Dash7 operates in the 433 MHz band, supports nominal and maximum data rates of 28 kb/s and 200 kb/s respectively. Dash7 is able to cover distances in the order of hundred meters to a few kilometers [82]. Dash7 networks are specifically suited for low power consumption applications where data transmission is sporadic and operated considerably slower such as telemetry systems [83]. Dash7 utilizes Bursty, Light, Asynchronous, Stealth, and Transitive (BLAST), *i.e.*, Dash7 networking technology is especially appropriate to be used in bursty and light (packet sizes are maximized to 256 bytes) applications with asynchronous communication [81]. In addition, Dash7-based devices are inherently portable and upload-centric, thus, the devices are not required to be managed by fixed infrastructure such as base stations [84]. Dash7-based devices are being used today in a vast number of applications such as building automation, smart meters, hazardous material monitoring, manufacturing and warehouse optimization, inventory management and mobile payments [85].

#### 2.2.11. ONE-NET

ONE-NET [86] is a proprietary open source standard, mainly designed to solve the problems of a wireless network in the home environment. ONE-NET is specifically optimized to support low-power long-range applications. One of the main characteristics of ONE-NET is that it is open to most proprietary software and hardware and is capable of being implemented with a vast range of low-cost low-power off-the-shelf microcontrollers and transceivers from numerous manufacturers such as Texas Instruments, Silicon Labs and Freescale. ONE-NET operates in different frequency ranges of 433 MHz, 868 MHz, 915 MHz and 2400 MHz. It uses Wideband FSK modulation technique and supports base and maximum data rates of 38.4 kb/s and 230 kb/s respectively. ONE-NET takes advantage of different network topologies for connecting ONE-NET-based devices. It utilizes peer-to-peer (P2P), star and multi-hop topologies with the master node organizing the P2P connections. Star topology is able to minimize cost and complexity of peripherals. Multi-hop network topology utilizes two or multiple wireless hops in order to cover larger communication area. ONE-NET is able to support maximum indoor and outdoor communication ranges of 100 m and 500 m respectively. ONE-NET wireless technology is specifically optimized for low-power consumption such as battery-operated devices. According to ONE-NET specification, low-duty-cycle battery-operated ONE-NET-based devices are able to achieve up to five years battery life on an AA or AAA Alkaline battery. In terms of security, ONE-NET utilizes the extended tiny encryption algorithm version two with thirty-two iterations [77].

#### 2.2.12. EnOcean

EnOcean [87] is a proprietary energy harvesting wireless sensor technology designed to be applied in a vast variety of applications such as building automation and control systems, transportation, cold-chain management, environmental monitoring and health monitoring. EnOcean is promoted by EnOcean Alliance to ensure interoperability of EnOcean products among different device vendors. EnOcean wireless technology is specifically optimized to provide solutions for ultra-low power consumption and energy harvesting applications. EnOcean-based devices utilize ultra-low power electronics and micro energy converters to enable wireless communications among battery-free sensors, switches, controllers and gateways. The main purpose of EnOcean’s energy harvesting technology is to derive energy from surroundings such as light, motion, pressure and transform them into electrical energy that can be utilized. Recently, EnOcean is ratified as a new international wireless standard by the International Electrotechnical Commission (IEC) as ISO/IEC 14543-3-10 to accelerate the development of energy-optimized wireless sensor networks. Products based on EnOcean are designed to operate without batteries and are engineered to run maintenance-free. EnOcean operates in frequency ranges of 315 MHz, 868.3 MHz and 902 MHz. It uses Amplitude-Shift Keying (ASK) modulation technique, utilizes relatively small data packets (limited to 14 bytes) and supports data rates up to 125 kb/s. This technology is also able to support maximum indoor and outdoor communication ranges of 30 m and 300 m respectively.

#### 2.2.13. Emerging Intra-Body Communication Technologies

Intra-Body Communication (IBC) technology is one of the emerging possible solutions for providing an ultra-low power communication over very short range links that specifically target WBAN applications. This technology is a non-RF wireless communication that utilizes human body as the medium for data transmission. IBC has recently been outlined in the newly ratified IEEE 802.15.6 standard and has shown to have advantages in terms of energy efficiency over many existing low-power RF protocols as it is able to transmit data with ultra-low transmission power below 1 mW [88,89].

The IBC technology utilizes three main approaches to wirelessly interconnect in-body implanted devices: ultrasonic communication, capacitive coupling and galvanic coupling techniques. Ultrasonic communication has recently been proposed in [90] to address the limitations of RF propagation in the human body. In water-based environments such as the human body where 65 percent is composed of water, radio waves are not perfectly suited. This is mainly due to the fact that water typically absorbs some portion of the radio waves. Thus, more amount of energy is required to successfully transfer the RF signal in the human body.

Hence, acoustic waves are considered one of the possible transmission technologies of choice for in-body communications as they are recognized to enhance the data throughput in media mostly composed of water comparing to RF signals [91]. Furthermore, Federal Communication Commission (FCC) regulations has limited the maximum allowable bandwidth that can be used for RF electromagnetic wave propagation available to Implanted Medical Devices (IMD) [91]. This therefore has greatly limited the data throughput of such devices [91]. As an example, for frequency range of 401–406 MHz, the maximum allowable bandwidth that can be used is around 300 kHz which greatly limits the communication rates of such devices to a maximum of 50 kb/s [91]. Alternatively, Okunev *et al.*, showed in [92] that digital acoustic intra-body systems can provide up to 0.5–1.0 Mbit/s data rate at BER = 0.001 with real acoustic transducers in frequency range of about 1–2 MHz [92].

In the capacitive coupling technique, human body is capacitively coupled to the surrounding environment [88]. In this technique, a current loop through the external ground creates the signal between the body channel transceiver. Alternatively, galvanic coupling method is performed by coupling Alternating Current (AC) in to the human body. In this technique, AC current is flowed through the body and human body is considered as a waveguide [88,89].

The energy efficiency advantage of these two coupling techniques over wireless protocols is mainly due to two reasons. One is due to the existence of lower path loss which does not include the otherwise detrimental effects of body shadowing in RF communications. The other reason is due to the utilization of wearable electrodes that are used as communication interface rather than low-impedance antennas. Moreover, in terms of security, IBC technology was shown itself to be more secure and less susceptible to interference compared to RF communication which makes it a possible low-power communication solution for Body Area Network (BAN) applications.

**Figure 2:**
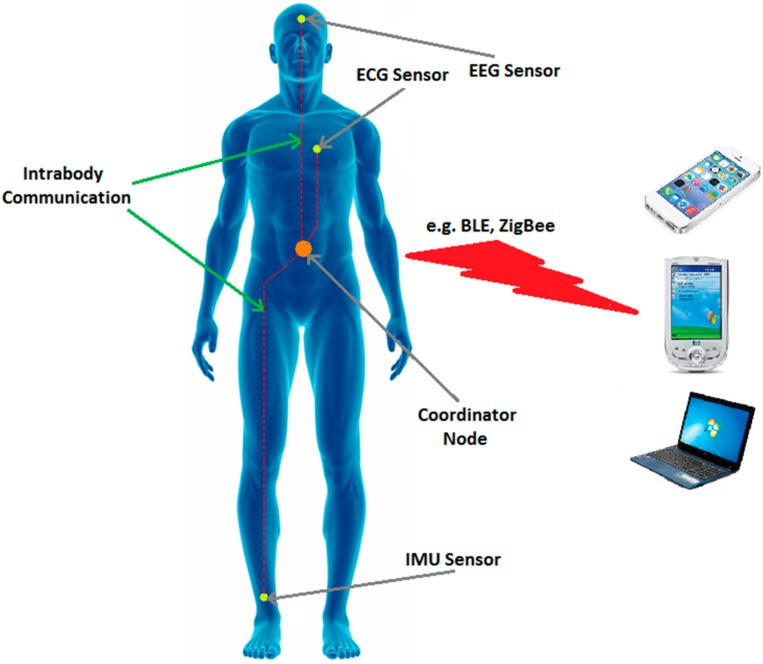
Intra-body communications combined with existing low-power protocols.

Nevertheless, IBC technology cannot solely be used in BAN systems. The data gathered by IBC-based sensors are required to be transmitted to a base station for further processing. For this reason, IBC technology must be combined with one of the existing energy efficient communication protocols such as ZigBee or Bluetooth Low Energy (BLE) [93]. Figure 2 shows a typical architecture of a possible energy efficient BAN system. In this scenario, IBC technology is employed for intra-body communications. IBC based sensor devices transfer the health-relevant information through the body to a central node which acts as a coordinator. This central coordinator is in charge of establishing a communication link between on-body devices and a base station. Thus, it uses one of the existing low-power communication protocols to transfer the collected data to a base station.

## 3. Discussion

In wearable health monitoring systems, energy efficient functioning of wearable devices is highly dependent on the selection of appropriate communication protocols. This is because wireless communication, unlike sensing and computation, consumes a significant amount of energy in the sensor nodes. Thus, a suitable selection of low-power communication technology can substantially increase the useful lifetime. This section highlights some of the important features of possible low-power communication technologies that must be taken into account when choosing a particular technology choice. There are a number of low-power wireless communication protocols that can accomplish this task. Out of these protocols, ZigBee and Bluetooth are most broadly used. The preference of Zigbee over Bluetooth/BLE or vice versa can be made based on the following factors. See Table 2, Table 3, Table 4 and Table 5 for a detailed comparison between ZigBee and BLE.

### 3.1. Protocol Efficiency

Protocol efficiency needs to be considered before selecting a low-power communication protocol. It greatly influences the energy efficiency of the selected protocol. This is because an inefficient communication protocol spends the majority of its time transferring overhead information rather than transmitting the actual payload data. Thus, little data may be transferred over a fixed duration of time and devices transferring the information may quickly run out of power. The efficiency of protocols can be calculated based on the ratio of actual payload information to the total length of the data packets. It is therefore very easy to compute the protocol efficiency of ZigBee and BLE by considering their packet formats (see Table 3); BLE has protocol efficiency of 66%, whereas ZigBee has protocol efficiency of 76%. Although, the results show that ZigBee is more protocol efficient than BLE, in many low data-rate low-power health monitoring systems wearable sensor nodes are only required to partially utilize the total available payload space to transfer data, hence, lower protocol efficiency does not necessarily mean that a particular protocol is inappropriate.

### 3.2. User Flexibility

According to the Bluetooth Special Interest Group (SIG), the majority of the Bluetooth-based smartphones will support BLE by 2018. This will offer great flexibility to end users, as a BLE-enabled smartphone can potentially be utilized as an access point. ZigBee needs a ZigBee-enabled device as an access point (currently there are no mobile phones with ZigBee capabilities).

### 3.3. Communication Range

ZigBee is considered to be a wireless Local Area Network (LAN) technology, thus it covers a greater range, whereas BLE is a WPAN protocol and its range is more limited. In a typical health monitoring system, there are scenarios in which collected data is required to be transferred to an access point within a room distance. In these scenarios, both BLE and ZigBee are considered as suitable protocols. However, in scenarios where data needs to be transmitted to a local station located in the other side of the house, if no other home networking infrastructures such as WiFi, PLC or Ethernet is employed, ZigBee is regarded as the better solution, simply because BLE is unable to cover the required distance by itself.

### 3.4. Energy Efficiency

Without a proper, in-depth analysis of these protocols, very little can be derived in terms of their energy efficiency. However, comparing the characteristics of these protocols can provide an approximate estimation of their energy expenditures during data transmission. Multiple access schemes are one of the important features that need to be considered more carefully as these can affect the energy efficiency of protocols. BLE uses Frequency Division Multiple Access (FDMA) along with Time Division Multiple Access (TDMA) schemes, whereas ZigBee employs CSMA/CA scheme. FDMA/TDMA schemes are more suitable to be used on high-load networks as they share the communication channel more efficiently and fairly, but are inefficient at low-load networks as there is usually delay in channel access. While the CSMA/CA scheme is more appropriate to be employed at low-load networks as there is no delay in channel access, is inefficient at high-load networks as packet collisions may happen. For more comprehensive comparison of ZigBee and BLE, see Table 2, Table 3, Table 4 and Table 5.

Due to the limited range requirements of a residential environment eHealthcare system, full meshing capability of any wireless communication platform may not be necessary, however if no meshing is supported, the infrastructure must be extended so that the premises is fully covered. Alternatively, multi-hop based routing of wireless communication packets may provide the required range of the application, but this solution requires multiple nodes with adequate power budget.

Alternative low-power wireless technologies include ANT, but also include recently developed proprietary technologies. These technologies usually are very constrained solutions that provide extremely low power requirements at the expense of much reduced data rate or range of communication. Some of them offer the flexibility of variable data rate and hence power consumption, and can operate at a number of radio frequencies.

A few of these protocols such as RuBee, Zarlink and Dash7 are only able to operate on lower frequency bands. Lower frequency bands are less crowded with radio services and they are less exposed to external interference, hence they have lower likelihood of packet collisions which results in lower power consumption. In addition, operating on lower frequency bands come with an advantage of good signal penetration through a variety of materials including the human body, however, the required antenna size is larger than those used at higher frequencies.

Among the low-power protocols, only ZigBee uses DSSS whereas BLE, Bluetooth and Wavenis employ FHSS. Spread spectrum techniques are employed for a range of reasons including increasing resistance to unwanted interference and noise. DSSS radios are believed to operate better for large data packets in low to medium interference environments, while FHSS radios operate better for small data packets in high interference environments. Moreover, FHSS radios perform better indoors and in harsh multipath environments because frequency hopping techniques are able to manage multipath fading environments by hopping to new frequency channels [94].

In terms of robustness, ANT, RuBee and Z-Wave only use error detection schemes such as CRC or Longitudinal Redundancy Check (LRC) whereas the rest of the protocols take advantage of an additional Forward Error Correction (FEC) technique along with error detection schemes. Error detection schemes are used in two-way communication systems in which packet retransmission will be requested by the receivers if errors are detected in the received data. This error control technique offers high transmission reliability and very low system complexity and is able to protect the information against most possible error occurrences over a comparatively quiet channel. However, applying a simple error-detection-only technique can also have a severe disadvantage. In erroneous channels, if the level of noise increases such that there is a high possibility that packets have at least one error, then the channel will quickly be occupied with retransmissions. As a consequence, no new information will be transmitted and the system throughput will decrease, and ultimately approach zero (This drawback affects many latency-sensitive applications such as health monitoring systems). Therefore, a combination of CRC and FEC techniques that most of the protocols use in this survey can protect the information in various channel conditions.

Many of these protocols such as BLE, Bluetooth, ANT, Z-Wave, Wavenis, BodyLAN and Dash7 use GFSK modulation while a number of other protocols such as Zarlink, Insteon and ONE-NET employ FSK modulation. GFSK modulation is an improved version of the FSK in which the data must be filtered via a Gaussian filter prior to modulating the carrier. This leads to a narrower power spectrum of the modulated signal which results in higher transfer speed of data in the same channel bandwidth [95]. In addition, GFSK modulation has the potential to cover a greater communication range compared to FSK modulation [96].

All these protocols are equipped with at least one type of encryption or level of security. Some of these encryptions are strong while others are very limited and offer little protection. In addition, Bluetooth, RuBee, Dash7 and EnOcean provide alternative security engines within the same chip which may be beneficial in particular applications. Some applications require less stringent security, whilst others may be able to exploit the optional extra encryption methods at different times. It is difficult to mention which security technique is more appropriate as it is so application and regulatory dependent. However, the fact that all radios offer some method of securing the communication channel ensures a level of security.

Alternative radios may also provide benefits such as a flexible packet format (length) that may result in more efficient packing of data per transmission. Most of these radios such as ZigBee, Wavenis and Dash7 do not require additional infrastructure in order to fully cover a residential area; however, some of them such as BLE, Bluetooth, Sensium and Zarlink may require more infrastructure to support a greater range. For more comprehensive comparison of alternative low-power wireless technologies, see Table 6, Table 7, Table 8, Table 9, Table 10, Table 11, Table 12 and Table 13. Star symbol in the Table 6, Table 7, Table 8, Table 9, Table 10, Table 11, Table 12 and Table 13 refers to undefined information.

Different protocols offer various connection management schemes. It may be considered a disadvantage if a body-worn sensor node has to maintain link with specific infrastructure or other sensor nodes. It may be advantageous if a link can be made and broken at any point without severely affecting latency and power budget.

## 4. Future Prospects

Nowadays, smartphone devices are more pervasive, user-accepted and powerful than ever. A large proportion of people carry their smartphones with them all the time and thus the idea of simple and continuous connectivity is not inaccessible anymore [97]. Mobile health (termed as mHealth) technologies have also experienced a slight change in direction from wearing and/or implanting body sensors to carrying a powerful wireless device with multifunctional capabilities such as a smartphone [98]. Healthcare providers may soon be able to monitor and measure vital signs without the need of on-body and/or implanted sensors (non-contact vital sign monitoring) [99]. For example, researchers from Rice University have been developing a non-contact video-camera system that can precisely monitor and measure temperature, pulse and breathing rate from changes in a patient’s skin color [99]. Smartphones can also be independently used for sleep monitoring. For instance, iSleep [100] takes advantage of the built-in microphone to detect the unconscious actions during sleep such as body movement, coughing and snoring, which are closely associated with the perceived quality of sleep people receive. Although, a more complete review of non-contact vital sign monitoring systems is not in the scope of this article, a number of examples of such systems are described in the literature [101,102,103]. The rest of this section is categorized into three parts. Part A summarizes a number of major advantages of smartphone-based healthcare applications. Part B considers some challenges of such solutions and finally part C explains the most areas of mHealth research that are expected to grow in the near future.

### 4.1. Advantages of Smartphone-Based Healthcare Applications

A collection of different types of low-cost sensors (e.g., accelerometer, gyroscope, camera, magnetometer, pedometer, goniometer, actometer, biometric and pressure) embedded in smartphones have enabled these multifunctional devices to be applied in many aspects of future healthcare systems. In addition, combination of some of these sensors such as biometric sensors with big data has provided a potential for smartphones to hugely impact the future of healthcare systems. For instance, people may habitually check their smartphones 100 times a day. This statistic information can be used to enable smartphone devices to frequently obtain the user’s facial scan. In this way, vital signs such as heart rate or blood pressure can be measured [104]. If this technique is used over a large population and such biometric data is collected in the cloud, contagious disease outbreaks can be discovered more quickly [104].

With the prevalent use of smartphones and the appearance of fourth generation of mobile telecommunications technology (4G) that provides higher speed mobile broadband internet access services along with the ubiquity of Wi-Fi technology, healthcare informatics (an interdisciplinary field combining healthcare, computer science and information science) is now able to overcome time and location limitations. This is enormously important specifically in cases that an immediate response is extremely critical or when a patient’s condition is not stable and dynamically changing.

In contrast to intrusive wearable devices that impose a burden on user’s daily activities, smartphones are non-intrusive, non-obstructive and not required to follow a cumbersome usage protocol. This results in reducing the possible usability complications.

Smartphones do not require supplementary hardware and many health-related mobile apps are accessible and free which lead to a more cost-effective solution compared to traditional wearable devices. Smartphones also have potential to manage chronic diseases such as Alzheimer’s, Hypertension and Diabetes. This can be done by frequent monitoring of patients through mobile apps or message reminders regarding the drug dosage information.

### 4.2. Challenges of Smartphone-Based Healthcare Applications

There is an uncertainty regarding the usefulness of disease control by smartphones. Ryan *et al.* [105] considered the cost-effectiveness of utilizing smartphone-supported self-monitoring of Asthma. He discovered that self-management by smartphones were not cost-effective in patients. This means that specific patient group will require careful, personalized treatment plan to address the specific needs and problems of patients who are suffering from a particular disease [106].

While the use of smartphones present great opportunities to improve healthcare quality for patients with chronic conditions, yet there has not been an effective strategy to move from pilot studies to implementation in the wider population [107]. In addition, the care of the elderly possibly cannot simply rely on smartphones as elderly individuals may be visually impaired, unable to use their hands effectively or even, unable to use the technology at all.

### 4.3. Fastest Areas of mHealth Growth in the Near Future

Areas of mHealth that are expected to have the most growth potential in the near future are explained as follows [108].

Patient monitoring is expected to have the fastest area of growth in the near future. This is because it is capable to early detect and prevent potential diseases that may occur later in life. This also can help to significantly reduce the cost of healthcare systems.

Patient location tracking is estimated to have the second most area of mHealth growth in the near future. This is simply because the need to locate and track patients with chronic conditions such as Alzheimer’s and dementia is great and thus the number of possible platforms proposing such solutions are steadily increasing [108].

## 5. Summary of Recent Research Articles

One of the main goals of this paper is to provide a brief overview of the most recent technological advances in the area of eHealthcare systems where healthcare providers are able to remotely monitor patients through the state-of-the-art WBAN systems along with existing ICTs. Since this area of research is able to significantly affect the existing healthcare systems by reducing the current operational costs, it has attracted the attention of a large number of researchers and scientists during the past decade and as a result of that many promising prototypes have been designed and developed. This section attempts to consider some of the most recent scientific publications in the field of telemonitoring systems for elderly and chronically ill patients.

In order to find the most relevant research articles in this area of research, three scientific databases were used for this review paper. We used the IEEE Xplore Digital Library, the ACM Digital Library and the PubMed scientific databases. The survey is limited to recent articles no older than five years as the wireless technologies of concern were only adopted widely in this period. In order to select the related articles from a large number of papers appeared in the search results, the following specific objective criteria were used when we were examining the abstracts and the main body of the texts, (A) only articles consisting of on-body (including wearable) sensors that may or may not be considered along with off-body (ambient) sensors; (B) articles that are more focused on elderly health monitoring and addressing chronic health issues; (C) articles that use a type of wireless communication technology. In addition, this survey excluded scientific papers that mainly address in-body (implantable) sensors and ambient sensors (out of the scope of this survey). The research selected 35 articles out of the 134 results that met the selection criteria. The main information extracted from these 35 articles is presented in Table 14.

### Survey Results

Among the articles that are included in Table 14, seven articles consider off-body (ambient) sensors along with on-body sensors [118,119,127,137,140]. In many of these articles, a data fusion technique is used to integrate multiple data sources into meaningful information. The other 28 articles investigate only on-body sensors [109,110,111,112,113,114,115,116,117,120,121,122,123,124,125,126,128,129,130,131,132,133,134,135,136,141,142]. Moreover, half of the articles employ mobile devices such as smart phones or PDAs as base stations. 16 articles out of 35 articles used classic Bluetooth as the main wireless communication technology [109,110,111,112,113,122,123,128,130,131,133,138,139,141,142,143]. Therefore, classic Bluetooth is considered as the most popular technology among the included articles. On the other hand, 14 studies used ZigBee as the main wireless communication technology [114,115,116,118,120,121,124,125,126,127,132,133,134,135,137,139]. Therefore, ZigBee is considered as the second most popular technology among the included articles. However, surprisingly, in none of the included studies BLE is used, which is likely to be explained because BLE is a relatively recent technology.

Five articles out of 35 articles as shown in [110,111,121,127,129] focus on fall detection systems based on various sensor types and different techniques, whereas, four articles of the articles that are listed in Table 14 [111,113,119,142] concentrate on Activities of Daily Living (ADL) of patients and elderly people. A few of these articles also investigate specific chronic conditions such as anxiety [112], chronic obstructive pulmonary and chronic kidney [138] and Parkinson’s disease [109,116].

Thirsty articles out of 35 articles that are listed in Table 14 used one of the popular wireless technologies such as Bluetooth or ZigBee. Some of the systems reviewed in Table 14 as shown in [112,117,119,134,139,142] use WiFi protocol for wireless communication. However, according to many studies such as [142,143,144,145,146,147,148], WiFi technology has shown that it is more power-hungry than Bluetooth technology. Thus, these systems appear to have high power requirement especially when compared to home-based eHealthcare system requirements. When power is of little concern, the choice of wireless is less critical and designers usually choose ones they are familiar with, easy to implement or in certain scenarios ones which fit with existing infrastructure (WiFi, GSM, *etc.*). A home environment eHealthcare system has tight restrictions on power consumption, which therefore rule out many protocols including WiFi and classic Bluetooth, or even ZigBee. There is however a distinct lack of application of alternative low- power wireless technologies or even BLE technology. This may be due to aforementioned need to interface with infrastructure or other devices.

A true home-based patient monitoring system must be able to transparently monitor individuals in home environment over extended periods of time. In these systems, sensor power requirements are of utmost importance. Hence it is imperative that the employed wireless technologies have minimal power consumption. If the application and choice of communication technology allow, energy harvesting based operation has the potential to power the devices indefinitely. It is expected that in the years following this survey a large body of research will accumulate with systems utilizing devices that require no specific attention from those they monitor (*i.e.*, charging). These systems would therefore allow devices to be worn in everyday clothing and be operating continuously.

## 6. Conclusions

In this survey paper, a review of the currently available low-power wireless communication protocols that can potentially be employed in WBAN systems is provided. More specifically, this survey paper provides a review of the current research in the area of WBANs with a specific focus on low-power consumption, transmission reliability, latency, data rates and security. A comparison of various energy-efficient and reliable wireless communication protocols are provided. This survey paper also considers the requirements and challenges of WBAN systems in a typical eHealthcare system in order to explore how such systems are able to effectively communicate with the home infrastructure. The imposed restrictions and requirements of WBAN systems are pointed out. The shortfalls of various WBAN systems in the residential environments are diagnosed and discussed and also suggestions for developing more appropriate systems for residential eHealthcare are proposed. In this survey, WBANs are used to enable healthcare professionals to continuously monitor patients and elderly people in their own residential environments. In this way, abnormal conditions can be detected early which results in major improvements in the quality of patients’ lives. This survey then investigates the future prospects of eHealthcare systems which includes the advantages, challenges and the fastest areas of growth in the near future. Finally, this survey concludes with a brief review of a number of currently published articles in the area of eHealthcare systems.

## Figures and Tables

**Table 1:** Possible wired home networking technologies.

Characteristic	RS-485	CAN	Ethernet
Network Topology	Bus	Bus	Star
Theoretical Max Bandwidth	35 Mbit/s	1 Mbit/s	10 Mbit/s−100 Mbit/s
Practical Bandwidth	1 Mbit/s	1 Mbit/s	2 Mbit/s
Stack Size (Use of resources)	Light	Light Plus	Heavy
Management of Cabling	Complicated	Complicated	Straightforward

**Table 2:** Physical layer comparison of ZigBee and Ble.

Characteristic	ZigBee	Bluetooth Low Energy
Frequency Band	2400, 868, 915 MHz	2400 MHz
Bit Rate	20 Kb/s (868 MHz), 40 Kb/s (915 MHz), 250 Kb/s (2400 MHz)	1 Mb/s
Modulation Type	BPSK, O-QPSK	GFSK
Spread Spectrum Technology	DSSS	FHSS
Nominal TX Power	–32 dBm to 0 dBm	–20 dBm to 10 dBm
Receiver Sensitivity	–85 dBm	–70 dBm
Number of Physical Channels	27 channels: 16 channels in the 2450 MHz, 10 channels in the 915 MHz, 1 channel in the 868 MHz	40 channels in FDMA: 3 advertising channels, 37 data channels
Channel Bandwidth	2 MHz (5 MHz wasteful spectrum)	2 MHz (no wasteful spectrum)

**Table 3:** Link layer comparison of ZigBee and Ble.

Characteristic	ZigBee	Bluetooth Low Energy
Multiple Access Scheme	CSMA-CA, slotted CSMA-CA	FDMA, TDMA
Maximum Packet Size	133 Bytes	47 Bytes
Protocol Efficiency (ratio of payload to total packet length)	102/133 = 0.76 (76 Percent Efficient)	31/47 = 0.66 (66 Percent Efficient)
Error Control Method	ARQ, FEC	ARQ, FEC
CRC Length	2 Bytes	2 Bytes
Latency	<16 ms (beacon-centric network)	<3 ms
Identifiers	16-bit short address 64-bit extended address	48-bit public device address 48-bit random device address

**Table 4:** Network layer comparison of ZigBee and Ble.

Characteristic	ZigBee	Bluetooth Low Energy
Network Topology	P2P, Star, Cluster Tree, Mesh	P2P, Star
Single-hop/Multi-hop	Multi-hop	Single-hop
Nodes/Active Slaves	>65,000	Unlimited
Device Types	Coordinator, Router, End Device	Master, Slave
Networking Technology	PAN	PAN

**Table 5:** Comparison of other properties of ZigBee and Ble.

Characteristic	ZigBee	Bluetooth Low Energy
Authentication	CBC-MAC	Shared Secret
Encryption	AES-CTR	AES-CCM
Range	100 Meters	10 Meters
Implementation Size	45–128 KB(ROM) 2.7–12 KB (RAM)	40 KB (ROM) 2.5 KB (RAM)

**Table 6:** Physical layer comparison of Bluetooth, Ant, RuBee, Sensium, Zarlink and Insteon.

Characteristic	Bluetooth	ANT	RuBee	Sensium	Zarlink	Insteon
Frequency Band	2400 MHz	2400–2485 MHz	131 KHz	868 MHz, 915 MHz	402–405 MHz, 433–434 MHz	RF: 869.85, 915, 921 MHz Powerline: 131.5 KHz
Bit Rate	1–3 Mbps	1 Mbps	9.6 Kbps	50 Kbps	200/400/800 kbps	RF: 38.4 Kbps Powerline: 13.1 Kbps
Modulation Type	GFSK	GFSK	ASK, BPSK, BMC	BFSK	2FSK/4FSK	RF: FSK Powerline: BPSK
Spread Spectrum Technology	FHSS	No	No	No	*	No
Nominal TX Power	0/4/20 dBm	4 dBm	−20 dBm	−10 dBm	2 dBm	*
Receiver Sensitivity	−90 dBm	−86 dBm	*	−102 dBm	−90 dBm	−103 dBm
Number of Physical Channels	79	125	2	16	10 MICS, 2 ISM	*
Channel Bandwidth	1 MHz	1 MHz	*	200 kHz	*	*

* refers to undefined information.

**Table 7:** Link layer comparison of Bluetooth, Ant, RuBee, Sensium, Zarlink and Insteon.

Characteristic	Bluetooth	ANT	RuBee	Sensium	Zarlink	Insteon
Multiple Access Scheme	TDMA	TDMA	*	TDMA, FDMA	*	TDMA + Simulcast
Maximum Packet Size	358 bytes	19 bytes	128 bytes	*	*	Standard: 10 bytes Extended: 24 bytes
Error Control Method	CRC, FEC	CRC	CRC	CRC, FEC	CRC, FEC	CRC, FEC
Checksum Length	1-byte/2-byte	2-byte	1-byte	*	*	1-byte
Identifiers	48-bit Public Device	*	32-bit	*	*	24-bit Module ID

* refers to undefined information.

**Table 8:** Network layer comparison of Bluetooth, Ant, RuBee, Sensium, Zarlink and Insteon.

Characteristic	Bluetooth	ANT	RuBee	Sensium	Zarlink	Insteon
Network Topology	Piconet, Scatternet	P2P, Star, Tree, Mesh	P2P	Star	P2P	Dual-mesh (RF & Powerline), P2P, Mesh
Single-hop/Multi-hop	Multi-hop	*	*	Single-hop	*	Multi-hop
Nodes/Active Slaves	8	65,000 + 1	Unlimited	8 + 1	*	Unlimited
Device Types	Master, Slave	Master, Slave	Controller, Responder	Master, Slave	*	All are peers
Networking Technology	PAN	PAN	PAN	PAN	PAN	PAN

* refers to undefined information.

**Table 9:** Comparison of other properties of Bluetooth, Ant, RuBee, Sensium, Zarlink and Insteon.

Characteristic	Bluetooth	ANT	RuBee	Sensium	Zarlink	Insteon
Security	Optional Pre-Shared Key, 128-bit Encryption	AES-128 Data Encryption, Link Authentication	Optional AES Encryption, Private Key, Public Key	Public Key	*	Rolling Code, Public Key
Range	10 m	30 m On-Body Only	30 m	5 m On-Body Only	2 m In-Body Only	45 m(Outdoors)
Implementation Size	100 Kbytes (ROM), 30 Kbytes (RAM)	128 Kbytes (Flash)	0.5–2 Kbytes (SRAM)	48 Kbytes (RAM), 512 bytes (ROM)	*	3 Kbytes (ROM), 256 Bytes (RAM)
Certification Body	Bluetooth SIG	ANT + Alliance	None	None	None	Insteon Alliance
Proprietary	No	Yes	No	Yes	Yes	Yes

* refers to undefined information.

**Table 10:** Physical layer comparison of Z-Wave, Wavenis, BodyLan, Dash7, One-net and Enocean.

Characteristic	Z-Wave	Wavenis	BodyLAN	Dash7	ONE-NET	EnOcean
Frequency Band	868, 908, 2400 MHz	433, 868, 915, 2400 MHz	2400 MHz	433 MHz	433, 868, 915, 2400 MHz	315, 868, 902 MHz
Bit Rate	9.6/40 Kbps, 200 Kbps	4.8/19.2/100 Kbps	250 Kbps, 1 Mbps	28, 55.5, 200 Kbps	38.4, 230 Kbps	125 Kbps
Modulation Type	GFSK	GFSK	GFSK	FSK, GFSK	Wideband FSK	ASK
Spread Spectrum Technology	No	Fast FHSS	*	No	No	No
Nominal TX Power	−3 dBm	14 dBm (Max)	0 dBm	0 dBm	*	6 dBm
Receiver Sensitivity	−104 dBm	−110 dBm	−93 dBm	−102 dBm	*	−98 dBm
Number of Physical Channels	*	16 Channels @ 433 & 868 MHz, 50 Channels @ 915 MHz	1	8	25	*
Channel Bandwidth	*	50 kHz	*	216, 432, 648 kHz	*	280 kHz

* refers to undefined information.

**Table 11:** Link layer comparison of Z-Wave, Wavenis, BodyLan, Dash7, One-net and Enocean.

Characteristic	Z-Wave	Wavenis	BodyLAN	Dash7	ONE-NET	EnOcean
Multiple Access Scheme	CSMA/CA	CSMA/TDMA, CSMA/CA	TDMA, CDMA	CSMA/CA	*	CSMA/CA
Maximum Packet Size	64 bytes	*	62 bytes	256 bytes	5 bytes	14 bytes
Error Control Method	LRC	FEC, Data Interleaving, Scrambling	CRC, FEC	CRC, FEC	*	CRC, FEC
Checksum Length	1-byte	No	*	2-byte	*	1-byte
Identifiers	32-bit (home ID), 8-bit (node ID)	48-bit MAC Address	*	EUI-64	*	*

* refers to undefined information.

**Table 12:** Network layer comparison of Z-Wave, Wavenis, BodyLan, Dash7, One-net and Enocean.

Characteristic	Z-Wave	Wavenis	BodyLAN	Dash7	ONE-NET	EnOcean
Network Topology	Mesh	P2P, Star, Tree, Mesh, Repeater	P2P, Ad-Hoc, Star	BLAST, Mesh	P2P, Star, Mesh	P2P, Star, Mesh
Single-hop/Multi-hop	Multi-hop	Multi-hop	*	Multi-hop	Multi-hop	Multi-hop
Nodes/Active Slaves	232	Up to 100,000	*	2^32^	4096	>4000
Device Types	Controller, Slave	Single Type	Single Type	Blinker, Endpoint, Gateway, Subcontroller	Master, Slave	Master, Slave
Networking Technology	PAN	LAN	PAN	PAN, LAN	PAN	PAN

* refers to undefined information.

**Table 13:** Comparison of other properties of Z-Wave, Wavenis, BodyLan, Dash7, One-net and Enocean.

Characteristic	Z-Wave	Wavenis	BodyLAN	Dash7	ONE_NET	EnOcean
Security	128-bit AES Encryption	128-bit AES Encription	*	Private Key (*i.e.*, AES 128), Public Key (*i.e.*, ECC, RSA)	XTEA2 Algorithm, Key Management	Rolling Code, 128-bit AES Encription, CMAC Algorithm, Private Key, Public Key
Range	30 m (Indoors), 100 (Outdoors)	200 m (Indoors), 1000 m (Outdoors)	122 m (Outdoors)	2000 m	100 m (Indoors), 500 m (Outdoors)	300 m (Outdoors), 30 m (Indoors)
Implementation Size	32–64 Kbytes (Flash), 2–16 Kbytes (SRAM)	48 Kbytes (Flash), 400 Bytes (RAM), 20 Bytes (Non-Volatile Memory)	*	8–16 KB (Built Size)	16 K (ROM), 1 K (RAM), 128 Bytes (Non-Volatile Memory)	32 KB (Flash), 2 KB (RAM)
Certification Body	Z-Wave Alliance	Wavenis Alliance	None	Dash7 Alliance	ONE-NET Alliance	EnOcean Alliance
Proprietary	Yes	No	Yes	No	No	No

* refers to undefined information.

**Table 14:** Included published articles between 2010 and 2015.

{Publication Date} [Ref.]	On-body OR Off-Body Sensors	Monitoring Parameters	Wireless Comm & Gateway	Novelty
{2015} [109]	On-body	Body Positioning, Motion	Bluetooth, Smartphone	A wearable assistant for gait training for Parkinson Disease with Freezing of Gait
{2015} [110]	On-body	Body Positioning	Bluetooth, Smartphone	A wristband community alarm with in-built fall detector
{2015} [111]	On-body	Body Positioning	Bluetooth, PC	Presents a description of the dataset for simulation of falls, near-falls and ADL
{2014} [112]	On-body	Spontaneous Blink Rate, Heart Rate	Bluetooth, Wi-Fi, PC	Anxiety detection technique using Google Glass
{2014} [113]	On-body	Skin Humidity, Heart Rate, Temperature, Body Positioning	Bluetooth, PC	Monitors ADL based on custom-designed wearable WSN
{2014} [114]	On-body	Body Positioning, Motion	ZigBee, PC	A low-cost open architecture wearable WSN for healthcare applications
{2014} [115]	On-body	Body Positioning	ZigBee, PC	Presents synchronous wearable WSN composed of autonomous textile nodes
{2014} [116]	On-body	Body Positioning, Motion	ZigBee, PC	A Parkinson’s Disease remote monitoring system based on WSN
{2014} [117]	On-Body	Heart Rate, Body Temperature	Wi-Fi, Smartphone, PC	Presents a system for remote monitoring based on mobile augmented reality (MAR) and WSN
{2013} [118]	On-body Off-body	Blood pressure, heart Rate, blood oxygen saturation, heart rate, body temperature, body positioning, pressure, humidity, carbon dioxide, explosive gas, ambient light, ambient temperature	ZigBee, Femtocell	Proposes a smart hybrid sensor network for indoor monitoring using a multilayer femtocell
{2013} [119]	On-body Off-body	Heart Rate, Body Positioning, Motion, sound	WiFi, GPRS, PDA, Smartphone	Proposes general rules of design of complex universal systems for health and behavior-based surveillance of human
{2013} [120]	On-body	Body Positioning	ZigBee, Sink Node	Focused on recognizing advanced motions (11 motions) by using 3D acceleration sensor
{2013} [121]	On-body	Body positioning (accelerometer & gyroscope)	ZigBee, Sink Node	A new fall detection system is proposed by using one sensor node which can be worn as a necklace
{2013} [122]	On-body	Heart rate, blood pressure, respiration rate, oxygen saturation	Bluetooth, GSM, Smartphone (Android-Based)	Reports preliminary study results that characterize the performance, energy, and complexity attributes of both mobile and cloud-based solutions for medical monitoring
{2012} [123]	On-body	Heart rate (PPG), body temperature, body positioning	Bluetooth, GSM, Smartphone	Monitors the posture of the patient in the bed (tilt monitoring) in order to help to reduce the cases of bedsore in bedridden elders
{2012} [124]	On-body	Atmospheric air pressure	ZigBee, PC	Presents a new approach to identifying and verifying the location of wearable wireless sensor nodes placed on a body by inferring differences in altitudes using atmospheric air pressure sensors
{2012} [125]	On-body	Heart rate, blood oxygen, body temperature, respiration rate, pulse rate	ZigBee, GPRS, Smartphone (Android-Based)	Proposes a new approach to monitor patients based on distributed WBAN
{2012} [126]	On-body	Unknown	ZigBee, GSM, PDA	Evaluates different types of interferences and disturbances such as ISI, MUI and noise through different techniques such as MUD receivers, DES-CMA and link adaptation
{2011} [127]	On-body Off-body	Body positioning, audio sound, motion difference with audio sound?	ZigBee, PC	Audio data processing and sound directionality analysis in conjunction to motion information and subject’s visual location is used to verify fall and indicate an emergency event
{2011} [128]	On-body	Heart rate, body positioning	Bluetooth, GSM, Smartphone	This work presents a methodology for an appropriate monitoring of strength training. The results are translated into appropriate feedback to the user
{2011} [129]	On-body	Body positioning, body pressure	Unknown, PC	Uses a waist-worn sensor for reliable fall detection and the determination of the direction of a fall
{2011} [130]	On-body	Heart rate, body temperature, blood oxygen, body positioning,	Bluetooth, GSM, Smartphone (Android-Based)	Textile platform based on open hardware and software, collects on-body data and stores them wirelessly on an open Cloud infrastructure
{2011} [131]	On-body	Heart rate, blood oxygen, body temperature, body pressure	Bluetooth, GSM, PC	The proposed system is a compact device which has various wearable sensors all attached inside a glove which continuously monitors vital parameters of the elderly person
{2011} [132]	On-body	Heart rate, blood pressure, temperature, blood oxygen	Proprietary, GSM, Smartphone	Shows how a group key can be securely established between the different sensors within a BAN
{2011} [133]	On-body	ECG, heart rate, respiration rate, body positioning	Bluetooth, GSM, PC, Smartphone	Proposes a system consists of a T-shirt sensorized to continuously record and analyzed human parameters during work activities at home
{2011} [134]	On-body	Heart rate, respiratory rhythm, oxygen saturation, blood pressure, body temperature	ZigBee, WiFi, GSM, GPRS, PDA	Proposes a system suitable for continuous long-time monitoring, as a part of a diagnostic procedure or can achieve medical assistance of a chronic condition
{2011} [135]	On-body	ECG	ZigBee, PC	Presents the development of a system for wireless ECG monitoring
{2011} [136]	On-body	ECG, blood pressure, heart beat rate, body temperature	Proprietary, GPRS, GSM, PC	Proposes a network based Wireless patient monitoring system, which can monitor multiple patients in hospital to measure various physical parameters
{2010} [137]	On-body Off-body	ECG, pressure, fire, light, moisture, sound, temperature	ZigBee, Laptop, PDA	A mixed positioning algorithm (object proximity positioning, signaling active positioning and signaling passive positioning
{2010} [138]	On-body Off-body	Heart rate, respiration, inspiration & expiration time & volume, temperature & humdity, motion activity & fall detection, cough & snoring detection, ambient light, carbon monoxide, volatile organic compound, air particle	Bluetooth, PDA	Addresses two specific diseases (chronic obstructive pulmonary disease and chronic kidney disease)
{2010} [139]	On-body Off-body	Heart rate, skin temperature, pulse rate, motion, physical contact	Bluetooth, WiFi, ZigBee, Z-Wave, GSM, IP, Home Base Station (with Hydra middleware)	Hydra middleware is used to make it possible to achieve integration and self-organization of sensors
{2010} [140]	On-body Off-body	Body positioning, motion	Unknown, PC	Applies real-time target extraction and a skeletonization procedure to quantify the motion of moving target
{2010} [141]	On-body	Heart rate, blood pressure, body positioning, location (GPS)	Bluetooth, GSM, GPRS, Smartphone, PDA	This system contains some functions to assist elderly such as regular reminder, quick alarm, medical guidance
{2010} [142]	On-body	Body positioning	Bluetooth, 3G, GPRS, WiFi, Smartphone (Windows based), PDA	Monitors the activity of individuals at night, through the use of simple wearable accelerometers
{2010} [143]	On-body	ECG, body temperature	Bluetooth, GSM, PDA, Smartphone	Designs a periodic data management system to manage wireless interface of sensor units with the patient database
